# Paneth Cells during Viral Infection and Pathogenesis

**DOI:** 10.3390/v10050225

**Published:** 2018-04-26

**Authors:** Mayumi K. Holly, Jason G. Smith

**Affiliations:** Department of Microbiology, University of Washington, Box 357735, 1705 NE Pacific St., Seattle, WA 98195, USA; mkh24@uw.edu

**Keywords:** Paneth cell, defensin, virus, regulated secretion, dense core vesicles

## Abstract

Paneth cells are major secretory cells located in the crypts of Lieberkühn in the small intestine. Our understanding of the diverse roles that Paneth cells play in homeostasis and disease has grown substantially since their discovery over a hundred years ago. Classically, Paneth cells have been characterized as a significant source of antimicrobial peptides and proteins important in host defense and shaping the composition of the commensal microbiota. More recently, Paneth cells have been shown to supply key developmental and homeostatic signals to intestinal stem cells in the crypt base. Paneth cell dysfunction leading to dysbiosis and a compromised epithelial barrier have been implicated in the etiology of Crohn’s disease and susceptibility to enteric bacterial infection. Our understanding of the impact of Paneth cells on viral infection is incomplete. Enteric α-defensins, produced by Paneth cells, can directly alter viral infection. In addition, α-defensins and other antimicrobial Paneth cell products may modulate viral infection indirectly by impacting the microbiome. Here, we discuss recent insights into Paneth cell biology, models to study their function, and the impact, both direct and indirect, of Paneth cells on enteric viral infection.

## 1. Location and Secretory Function of Paneth Cells

Paneth cells are located at the base of the crypts of Lieberkühn in the small intestine of various animals and are interspersed amongst the intestinal stem cells from which they differentiate [1,2,3,4,5,6,7,8]. Unlike other differentiated epithelial cell types (goblet cells, enteroendocrine cells, tuft cells, and enterocytes), which migrate out of the intestinal crypt, Paneth cells remain within the crypt base [9]. Also, unique to Paneth cells are their long lifespans (≈30 days) compared to the other differentiated epithelial cell types (≈3–5 days) [10,11,12]. Paneth cells are characterized by apically located, large, cytoplasmic, and electron-dense granules that contain a mixture of antimicrobial peptides and proteins, cytokines, scaffolding molecules, and proteases. Mature granules are surrounded by an acidic mucopolysaccharide complex, which is visible as a halo in electron micrographs [13]. Like many dedicated secretory cells that undergo regulated secretion, Paneth cells package only a subset of their secreted proteins into their cytoplasmic granules. They also utilize constitutive secretion mechanisms common to most epithelial cells. Because of a lack of long-term culture models for Paneth cells that have only been recently resolved (see Section 7 below), the mechanism by which Paneth cells specifically package contents into their granules remains poorly understood. However, general models of regulated vs constitutive secretion in other cell systems likely also apply to Paneth cells [14,15,16]. A series of studies have elucidated secreted products, both granule-dependent and independent, that are specific to Paneth cells through a variety of methods including laser capture microdissection and single cell transcriptomics [1,3,7,17,18,19,20,21,22]. The most well-known and characterized granule contents are the antimicrobial peptides and proteins; however, other proteins have also been localized to these structures. A subset of notable granule constituents is discussed below.

## 2. Granule Contents (Regulated Secretion)

***Antimicrobials***. Several types of antimicrobial peptides and proteins are packaged into and secreted via Paneth cell granules including enteric α-defensins, lysozyme, secretory phospholipase A2 (sPLA_2_), angiogenin-4 (Ang4), RegIIIγ, and α1-antitrypsin. Collectively, these molecules have broad antimicrobial activity against a wide range of organisms. Enteric α-defensins are the most abundant secreted product [23,24]. Additionally, of the antimicrobial products packaged into Paneth cell granules, only α-defensins have known anti-viral activity, which has been recently reviewed [25,26]. α-defensins are small, cationic, and amphipathic peptides with two β-sheets stabilized by three disulfide bonds [25,27,28]. Humans encode genes for two α-defensins, human defensin 5 (HD5), and human defensin 6 (HD6), whereas mice encode over 25 different enteric α-defensin genes, although only a subset is expressed abundantly in any given mouse strain [29]. The broad antimicrobial activity of HD5, including potent antiviral activity, is well documented [25,26,30,31], while HD6 has a unique mode of action and functions by trapping bacteria and fungi [32].

The other antimicrobial constituents of the Paneth cell granules are not known to be active against viruses. Lysozyme is an enzyme that cleaves peptidoglycan in the cell walls of bacteria [33]. Mice encode two genes for lysozyme; one is expressed in Paneth cells and the other is expressed in macrophages [34]. In contrast, humans encode only one lysozyme gene that is expressed in both Paneth cells and macrophages. RegIIIβ and RegIIIγ (HIP/PAP in humans) are antibacterial C-type lectins that target peptidoglycan of Gram-positive bacteria [21,35]. Human and mouse sPLA_2_ catalyzes the hydrolysis of phospholipids and is bactericidal for Gram-positive, but not Gram-negative, bacteria [36,37]. Mouse Ang4 is a bactericidal member of the RNase superfamily with activity against both Gram-positive and Gram-negative bacteria, although the human ortholog angiogenin is not localized to Paneth cells [20]. α1-antitrypsin is a serine protease inhibitor with some antimicrobial activity including inhibition of hemolysis by enteropathogenic *Escherichia coli* and *Cryptosporidium parvum* infection [38]. A naturally occurring peptide derived from this protein also has anti-HIV activity [39]. Of these antimicrobial peptides and proteins, Paneth cells are the sole epithelial source within the intestine of α-defensins [40], lysozyme [41], sPLA_2_ [42], and Ang4 [20], while RegIIIγ, RegIIIβ, and α1-antitrypsin are also produced by other epithelial cell types [43,44].

***Cytokines***. Two cytokines have been identified in Paneth cell granules: interleukin (IL)-17A [19] and tumor necrosis factor-α (TNF-α) [45]. IL-17A, a molecule that was traditionally thought to be produced only by immune cells, was found specifically in Paneth cells using laser capture microdissection and has been localized to granules by immunoelectron microscopy [19]. IL-17A is an important factor during immune responses to invading pathogens through stimulation of antimicrobial peptide production and cytokine secretion [46]. Following hepatic ischemia and reperfusion injury, high amounts of IL-17A were detected in the serum and small intestines of treated mice [47]. Paneth cells were identified as the source of this IL-17A by a reduction in IL-17A levels in mice lacking Paneth cells due to deletion of *Sox9* in the intestinal epithelium.

TNF-α is typically produced by immune cells and functions to potentiate systemic inflammation. TNF-α mRNA was first identified in Paneth cells in 1990 [45] and was then localized specifically to the granules [48]. Endotoxin treatment of mice results in degranulation of Paneth cells and the subsequent release of TNF-α into the crypt lumen [48]. However, the exact role that Paneth cell-derived TNF-α plays in homeostasis and disease remains to be determined.

***Proteases***. A characteristic of regulated secretion is the need to package granule contents at high density and in forms that are not cytotoxic. To limit cytotoxicity, the granule contents of Paneth cells, like those of other cells that use regulated secretion, are often subject to post-translational activation. One common paradigm is the proteolytic cleavage of an inactive precursor into a mature, active form. This mechanism has been well-characterized for enteric α-defensins. In the mouse, matrix metalloproteinase 7 (MMP7) is co-packaged in Paneth cell granules and activates pro-defensins [49]. This cleavage can occur intracellularly, although packaged enteric α-defensins are not completely processed [50]. MMP7 also cleaves a related class of antimicrobial peptides, called cryptdin-related sequences peptides, that are unique to the mouse [51]. In humans, HD5 and HD6 are processed by Paneth cell trypsin [30]. Trypsin is packaged into granules as a zymogen and is activated through an unknown mechanism upon degranulation; thus, no intracellular processing of HD5 and HD6 occurs. There are three isoforms of trypsin that are produced in the pancreas, but Paneth cells appear to only produce two of the three isoforms. Remarkably, mouse Paneth cells do not appear to make their own trypsin, and *MMP7* is not expressed in the human intestinal mucosa.

## 3. Non-Granule Products (Constitutive Secretion)

In addition to their granule contents, Paneth cells also secrete proteins through constitutive secretion. These include innate immune molecules (e.g., interferon- β (IFN-β) and IL-1β) common to many epithelial cells as well as homeostatic cues essential for maintenance of the stem cell niche that are unique to Paneth cells: Wnt, epidermal growth factor (EGF), and Notch ligands [52].

Wnt proteins are membrane-bound ligands critical for development, cell migration, and cell polarization [53]. Within the intestine, Wnts are required for proper development and health of stem cells [54,55]. Several Wnts are expressed in the small intestine by Paneth cells and by non-epithelial stromal cells, including Wnt2b, Wnt3, Wnt4, Wnt5a, Wnt6, and Wnt9b [56]. However, only a subset of these (Wnt3, Wnt6, and Wnt9b) is expressed in isolated intestinal crypts, indicating they play a direct role in stem cell maintenance [56]. Wnt signaling in intestinal development and homeostasis has been studied most extensively in the mouse and is supported by in vitro studies in the recently developed enteroid model (see Section 7 below) [54,56,57]. Wnt signaling controls the expression of some Paneth cell-specific secretion products (e.g., MMP7, enteric α-defensins), transcription factors required for Paneth cell differentiation (SOX9), and signaling molecules that dictate proper positioning along the crypt/villus axis (EphB2 and EphB3) (see Section 6 below) [58].

EGF is important for crypt cell proliferation through activation of the ERK pathway. EGFR, the receptor for EGF, is expressed on intestinal stem cells [52,59]. Thus, close association of the EGF-producing Paneth cells and intestinal stem cells in the crypt base facilitates stem cell proliferation. However, the mitogenic potential of Paneth cell-derived EGF is checked by tight regulation of the EGF-ErbB pathway [59].

Paneth cells express Notch ligands, such as Dll1 and Dll4 on their membranes, which bind the Notch receptor on intestinal stem cells [52]. Notch receptor engagement results in activation of target genes important in intestinal stem cell homeostasis, such as *Hes1*. Inhibition of this signaling pathway either through small molecules or genetic knockout of *Hes1*, *Notch1*, or *Notch2* results in increased goblet cell differentiation in the mouse small intestine [60,61,62,63,64]. In contrast, activation of the Notch pathway results in increased proliferation in the stem cell compartment with a concomitant loss in secretory cell differentiation [60].

## 4. Mechanisms of Packaging

Two potential mechanisms that are not necessarily mutually exclusive have been proposed for sorting of specific cargo into dense core vesicles (DCVs) such as Paneth cell granules: sorting by entry and sorting by retention [65,66]. In the sorting by entry model, proteins must interact with a specific receptor to enter a budding DCV [65]. Sorting by entry can be further subdivided into two classes: sorting by aggregation and sorting by insertion of the protein into lipid rafts [67]. Sorting by aggregation occurs when proteins targeted to DCVs aggregate via a pH- and cation-dependent mechanism or are nucleated by scaffolding molecules [65,66,67,68]. The other subclass of sorting by entry entails insertion of a C-terminal domain directly into lipid rafts of the trans-Golgi network (TGN). In contrast, the sorting by retention model posits that all proteins are initially packaged into immature DCVs, but only specific proteins are retained [65,67]. Proteins not destined for DCVs are removed in constitutive-like vesicles; however, the basis for identifying which proteins are to be removed is not known for all proteins [67]. Studies using a variety of different cell types have found evidence for all of these mechanisms, suggesting that the method of sorting may be protein- or cell type-dependent [65,69].

Dense core vesicle formation begins with vesicle budding from the TGN [67,69]. Following release from the TGN, vesicles undergo a series of maturation steps to form mature DCVs as they traffic to the plasma membrane [69]. It has been proposed that two populations of DCVs exist in a secretory cell, a readily releasable pool (RRP), and a reserve pool (RP). The RRP is primed for release upon stimulation with a given ligand, whereas the RP requires several additional steps to be released (trafficking, docking, and priming). The applicability of these general models to Paneth cells has yet to be elucidated.

Lysozyme is the only Paneth cell granule constituent for which packaging has been worked out in detail. Two recent studies demonstrated a role for NOD2, RIP2, LRRK2, and RAB2a in this process [70,71]. NOD2 sensing of microbiome-derived peptidoglycan is necessary for lysozyme sorting into Paneth cell granules [71]. In the absence of either LRRK2 or RIP2 within the granule, lysozyme is initially packaged into immature granules in the subapical region but is not retained in the mature granules due to selective targeting for lysosomal degradation [70,71]. RAB2a, a protein involved in vesicle sorting in *Caenorhabditis elegans* [72], is recruited to Paneth cell granules in an LRRK2- and RIP2-dependent manner and is important for correct DCV trafficking [70,71]. Interestingly, enteric α-defensin packaging into Paneth cell DCVs was unaffected when this pathway was perturbed. In contrast, Paneth cell packaging and secretion can be altered more broadly during bacterial infection. Bel and colleagues found that infection of mice with *Salmonella enterica* serovar Typhimurium (STm) induces endoplasmic reticulum (ER) stress in Paneth cells, leading to accumulation of autophagosomes containing lysozyme [73]. Under these conditions, lysozyme was secreted through the secretory autophagy pathway. In addition, although not co-localized with lysozyme in vesicles, α-defensin packaging into Paneth cell granules was also perturbed. Therefore, further studies on the mechanisms of packaging of lysozyme and other granule contents, the maturation of DCVs in Paneth cells, and alternative secretion pathways under homeostatic and inflammatory conditions are needed.

## 5. Mechanisms of Secretion

Granule contents of Paneth cells appear to be regulated at the level of secretion rather than at the level of transcription or translation. However, there is some data for transcriptional regulation that contributes to differences in expression of specific paralogs of mouse α-defensins [74,75]. Quantitative analyses are complicated, because Paneth cell expansion, which has been shown to occur in response to infection and injury [76,77,78], would also manifest as apparent transcriptional upregulation. Additionally, an appropriate factor that is unique to Paneth cells has not been identified to normalize for cell numbers.

Two non-mutually exclusive modes of secretion, holocrine (cell extrusion from the intestinal epithelium) and merocrine (secretory granule release by exocytosis), have been put forth to explain Paneth cell degranulation [79,80,81]. Furthermore, there is conflicting data on the types of physiologic stimuli that trigger secretion. Merocrine secretion was initially posited as the sole mechanism of Paneth cell degranulation and is consistent with the long lifespan of Paneth cells in vivo [79]. In these experiments, crypts isolated from the small intestine of mice were exposed to bacteria and bacterial products, and antibacterial activity in the supernatant was measured to assess the stimulatory capacity of the ligands. From these experiments, it was concluded that Paneth cells degranulate in response to lipopolysaccharide (LPS), muramyl dipeptide, lipid A, lipoteichoic acid, and live bacteria. However, these experiments are complicated by the instability of purified intestinal crypts; once isolated, crypts rapidly undergo anoikis.

**Figure 1:**
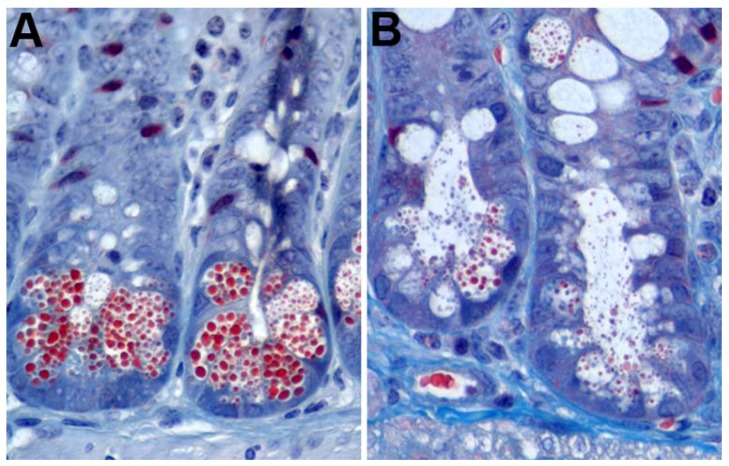
Mouse Paneth cell degranulation. (**A**) Trichrome staining of untreated mouse intestinal epithelium showing prominent Paneth cell granules (red); (**B**) mouse crypts 7 min after intraperitoneal injection with 10 µg/g aceclidine showing granule release into the crypt lumen.

Paneth cells also undergo merocrine secretion in response to cholinergic agents and specific cytokines [2,80,82,83,84,85,86]. The enteric nervous system is in close proximity with intestinal crypts, and stimulation of the epithelium triggers secretion by Paneth and goblet cells [87]. In conjunction with this phenomenon is the idea that eating stimulates Paneth and goblet cell secretion in order to prepare the intestine for the arrival of food and associated micro-organisms, which is consistent with the observation that Paneth cell granules accumulate in fasting animals [3,8,88]. Nervous stimulation can be modeled in vitro through the addition of cholinergic agents, chemicals that function as or mimic neurotransmitters, to cell cultures containing Paneth cells [79,80,89]. Moreover, treatment of mice with cholinergic agents, such as carbamylcholine and aceclidine, stimulates Paneth cells to degranulate [82] (Figure 1). Both in vitro and in vivo, crypts exposed to cholinergic agents still maintain morphologically distinct Paneth cells, but they contain fewer granules and large cytoplasmic vacuoles, which are suggestive of merocrine secretion. IL-4 and IL-13 treatment of small intestinal tissue explants induces degranulation without death of the Paneth cells, which is consistent with merocrine secretion [90]. Additionally, the model for merocrine secretion is in agreement with the concept of two separate pools of granules. Paneth cells secrete the RRP immediately following stimulation, which is then repopulated from the RP.

Recently, a second model of Paneth cell secretion was proposed in which Paneth cells die and extrude into the lumen upon IFN-γ treatment [80]. These authors found no evidence for the previously described bacterial product-dependent Paneth cell degranulation or extrusion. While IFN-γ-dependent Paneth cell extrusion is likely a physiologically relevant event [91,92], extrusion is unlikely the only mechanism of Paneth cell degranulation, since it requires Paneth cell death followed by regeneration of Paneth cells from intestinal stem cells and would be inconsistent with a long lifespan in vivo. Since compelling evidence exists for both models of Paneth cell secretion, it is possible that one operates primarily under homeostatic conditions (merocrine), while the other functions during inflammation (holocrine).

Zinc has long been known to be concentrated in Paneth cells [93,94]; however, the functional importance of zinc in Paneth cells was unknown until recently. ZnT2, a zinc transporter, localized specifically to Paneth cell granules [95]. Mice lacking ZnT2 exhibited abnormal Paneth cell granules. Moreover, loss of ZnT2 resulted in reduced bacterial killing by crypt secretions compared to wild-type secretions, suggesting that Paneth cell secretion was impaired by the lack of zinc.

## 6. Paneth Cell Development

***Paneth cell origins***. All of the intestinal epithelial lineages are derived from intestinal stem cells. Intestinal crypts contain two classes of multipotent intestinal stem cells: Lgr5^+^ crypt base columnar cells (CBCs) and +4 label retaining cells (LRCs) [52]. Lgr5^+^ CBCs undergo symmetrical division and stochastically become another CBC or a transit amplifying cell [96]. Of the subset of CBC daughter cells that become transit amplifying cells, some will differentiate into Paneth cells and remain in the crypt base. The remainder of the transit amplifying cells will migrate out of the stem cell compartment and differentiate into one of the other intestinal epithelial cell types along the villi. Recent studies have shown that the +4 LRCs are transit amplifying cells that are precursors of Paneth cells and enteroendocrine cells [97]. This population is not well-defined by markers (*Bmi1*, *mTert*, *Hopx*, and *Lrig1*), because expression of these markers is not exclusive to the crypt base [9]. However, lineage tracing experiments, as well as studies on sensitivity to radiation treatment, have identified a population of cells at approximately the +4 position that retain label for a prolonged period of time, are actively cycling, and are sensitive to radiation damage. Thus, under normal conditions, Paneth cells and enteroendocrine cells differentiate from CBCs via the +4 LRCs; however, there is evidence for plasticity in the intestinal compartment whereby the +4 LRCs and even more committed lineages such as enterocytes can revert to a more stem cell state when the CBCs are lost due to damage [97,98]. Inflammation and physical damage can also stimulate an expansion of the Paneth cell compartment [76,78].

***Signaling pathways involved in the expression of specific gene products***. Many of the Paneth cell-specific gene products are controlled by Wnt signaling [99,100,101]. When Wnt is present, it binds to its receptor Frizzled, leading to an intracellular signaling cascade that culminates in translocation of β-catenin to the nucleus and association with specific members of the T cell factor (TCF) family to act in concert to turn on transcription of target genes [52,53]. The promoters of enteric α-defensin genes in mice, *DEFA5* (HD5) and *DEFA6* (HD6) in humans, and *Mmp7* in both species contain consensus sequences for β-catenin/TCF-binding sites [99,101,102,103]. Co-transfection of luciferase reporters driven by mouse enteric α-defensin, *DEFA5*, and *DEFA6* promoters with activated β-catenin and TCF4 constructs greatly increased luciferase activity over basal activity [99]. Additionally, mutation of the β-catenin/TCF-binding sites in the *DEFA6* promoter significantly reduced luciferase activity of these constructs. Furthermore, deletion of *Tcf4* in the embryonic mouse small intestine significantly reduced enteric α-defensin expression [58]. Thus, the Wnt-β-catenin/TCF axis is important in regulating expression of specific Paneth cell products.

Although lysozyme is also packaged into Paneth cell granules, its promoter does not contain β-catenin/TCF-binding sites [58,99]. The specific signaling pathways involved in lysozyme expression in Paneth cells are difficult to parse from the pathways involved in Paneth cell differentiation, since lysozyme staining is often used to quantify Paneth cell numbers. Inhibition of PI3K in mouse enteroids results in increased lysozyme expression; however, this effect is likely mediated through expansion of the Paneth cell population [104].

Similarly, fibroblast growth factor receptor-3 (FGFR-3) signaling has also been implicated in the expression of Paneth cell-specific secretory products but also appears to affect Paneth cell development. *Fgfr^−^*^/*−*^ mice have fewer crypts, fewer Paneth cells per crypt, and reduced expression of lysozyme and mouse enteric α-defensin-5 [105]. Interestingly, although these mice had fewer Paneth cells, the Paneth cells that did develop looked morphologically normal. Moreover, treatment of a human colorectal cancer cell line (Caco-2) with FGFR-3 ligands resulted in increased expression of the Paneth cell specific markers HD5, HD6, and lysozyme, and increased TCF4/β-catenin activity [106]. Thus, FGFR-3 signaling plays an important role in Paneth cell differentiation and gene product expression.

***Signaling pathways involved in morphology***. In addition to unique gene expression, secretory morphology is a defining feature of Paneth cells. The genetic factors that contribute to this characteristic are not completely known, and there is evidence that signaling pathways that specify Paneth cell secretory morphology are distinct from those that control the expression of Paneth cell secretory products. For example, Wnt/β-catenin signaling is dysregulated in some tumors that express Paneth cell gene products without a secretory morphology [58]. Additionally, in mice, Paneth cell effectors are expressed prior to the development of mature Paneth cells, which begins 7 days after birth and reaches adult levels 30 days after birth [107,108,109].

Wnt signaling is critical for Paneth cell formation in addition to Paneth cell-specific gene expression [110]. Ectopic expression of Wnt3 in mouse enteroids (see Section 7 below) induces Paneth cell differentiation [56]. Conditional deletion of APC, a protein complex that prevents β-catenin translocation to the nucleus, in intestinal epithelial cells results in increased Paneth cell differentiation and mislocalization out of the crypt base in the small intestine and Paneth cell formation in the colon, which does not normally contain Paneth cells [100]. Conversely, expression of a hypomorphic allele of the β-catenin gene led to reduced numbers of granular cells in intestinal crypts and an associated decrease in production of lysozyme and Ang4. Abnormal Paneth cell localization in these models may be due to disruption of the EphB receptor/B-type ephrin gradient. EphB receptors are TCF/β-catenin responsive receptors required to correctly position Paneth cells along the crypt/villus axis [102]. *EphB3* is normally expressed in the crypt up to the +4 LRC position. Cells expressing this receptor are positioned inversely to a gradient of the ligand ephrin-B1, expression of which is decreased by TCF/β-catenin signaling [102,111]. Thus, deletion of *EphB3* also leads to mislocalization of Paneth cells outside of the crypt base [102]. Interestingly, these mislocalized Paneth cells lack nuclear β-catenin but express lysozyme, demonstrating that Paneth cell morphology and lysozyme expression can occur in the absence of β-catenin stabilization and translocation to the nucleus.

Several transcription factors are essential for the specification and differentiation of Paneth cells [110]. MATH1/ATOH1 is negatively regulated by Notch signaling [52] and is an essential transcription factor for differentiation of the secretory lineage [112]. Loss of *Math1* in vivo results in loss of Paneth cells, goblet cells, and enteroendocrine cells. Additionally, activation of PI3K by the neuregulin receptor ErbB3 negatively regulates *Math1*, controlling Paneth cell numbers in vivo [104]. Downstream of MATH1 is GFI1, a zinc finger transcriptional repressor [113]. Loss of *Gfi1* in mice results in a similar phenotype to *Math1^−^*^/*−*^ mice with a loss of Paneth cells and fewer goblet and enteroendocrine cells [113]. Another transcription factor important for Paneth cell development is SPDEF. *Spdef^−^*^/*−*^ mice do not have mature Paneth cells or goblet cells but do express markers of secretory lineage commitment in their intestines [114]. These mice also express higher levels of Dll1, a Notch ligand typically expressed on secretory progenitors, in their crypts, suggesting that SPDEF functions promote differentiation of Dll1^+^ cells into the secretory lineage. Thus, *Spdef* plays a role upstream of Paneth cell and goblet cell specification and prior to full Paneth cell differentiation. Finally, *Sox9*, a member of the Sox family of transcription factors, is a proximal factor required for Paneth cell development. Deletion of *Sox9* in intestinal epithelial cells results in loss of Paneth cells and enlargement of the intestinal crypts with no effect on other lineages [115,116]. In the absence of Paneth cells, proliferating cells occupied the base of the crypts [115].

***Autophagy and the unfolded protein response***. Due to their longevity, Paneth cells are sensitive to defects in the autophagy pathway, which is the regulated lysosomal degradation of organelles and cellular proteins that have been damaged by various cellular processes. Autophagy proteins ATG16L1, ATG5, and ATG12 form a complex that catalyzes microtubule-associated protein light chain 3 (LC3) lipidation [117]. ATG7 is critical for association of ATG5 and ATG12 [118]. Mice deficient in *Atg16l1*, *Atg5*, or *Atg7* exhibit reduced numbers of granules, increased numbers of cytoplasmic vacuoles, degenerating mitochondria, and diffuse cytoplasmic staining of lysozyme in their Paneth cells [22,119]. Interestingly, these phenotypes are dependent upon concomitant infection with murine norovirus (MNV), a member of the *Caliciviridae* family [120]. Mice lacking *Atg16l1* specifically in intestinal epithelial cells (*Atg16l1*^Δ*IEC*^) were also more susceptible to MNV-triggered epithelial damage with pronounced loss of Paneth cells upon dextran sulfate sodium (DSS) treatment [121]. This effect was likely mediated through a role of ATG16L1 in blocking necroptosis, a form of programmed cell death, in intestinal epithelial cells. Mice deficient for *Atg16l1* in their intestinal epithelial cells also show impaired responses to STm, notably decreased antimicrobial peptide expression, elevated inflammation, and increased bacterial translocation compared to wild-type mice [122]. However, mice expressing a hypomorphic allele of *Atg16l1* were not more susceptible to *Listeria monocytogenes* infection [22]. The discrepancy in these results could be due to the difference between Gram-negative and Gram-positive bacteria or the difference between expressing a hypomorphic allele and cleanly knocking out the gene in a specific cell lineage.

An additional protein, IRGM1 in mice (IRGM in humans), is potentially involved in autophagy and Paneth cell physiology. IRG proteins are similar to dynamins in their ability to control membrane fusion and vesicle trafficking, and IRGM1 has recently been linked to regulating autophagy [123]. *Irgm1^−^*^/*−*^ mice exhibit Paneth cell abnormalities similar to *Atg16l1^−^*^/*−*^ mice. The Paneth cell granules of these mice were abnormal in size with less dense granules. This reduction in density was associated with increased halos around the electron dense granules as visualized by transmission electron microscopy. Additionally, lysozyme-positive cells were found outside of the crypt, indicating dysregulation of Paneth cell localization. Notably, *Irgm1^−/−^* mice are more susceptible to ileal injury following DSS treatment compared to wild-type mice. This is unexpected, as DSS treatment typically causes disease in the colon and not the small intestine. An important caveat to these studies is the impact of the microbiome on the observed phenotypes. *Irgm1^−/−^* mice re-derived under specific pathogen free (SPF) conditions have only a modest increase in susceptibility to DSS treatment compared to SPF wild-type mice [124]. Additionally, goblet and Paneth cells did not exhibit any abnormal phenotypes. Since the bacterial communities differed significantly between the conventionally reared mice and the SPF mice, this is another example in which a combination of susceptibility genes and environmental factors determines the disease outcome.

The secretory function of Paneth cells demands significant protein synthesis and folding in the ER, making them vulnerable to ER stress. Several proteins involved in the unfolded protein response (UPR) have been implicated in Paneth cell function [117,125]. X box binding protein 1 (XBP1) is a transcription factor essential for the UPR. Crypts from *Xbp1^−^*^/*−*^ mice lack cells with electron dense granules [126] and have reduced lysozyme and enteric α-defensin staining, indicative of Paneth cell loss [125]. These mice undergo spontaneous enteritis [125,126], implicating *Xbp1* in initiating intestinal inflammation. Interestingly, *Xbp1* deletion specifically in Paneth cells resulted in spontaneous enteritis similar to mice in which *Xbp1* was deleted in all intestinal epithelial cells. Moreover, *Xbp1^−^*^/*−*^ mice were more susceptible to *L. monocytogenes* infection, which is likely due to the reduced bactericidal activity of Paneth cells, since crypt supernatants from *Xbp1^−^*^/*−*^ mice were unable to kill *L. monocytogenes* [125]. The UPR and autophagy pathways both function to modulate intestinal inflammation. Loss of key components of both pathways (XBP1 and ATG16L1) resulted in ileitis more severe than with the loss of either protein alone [126]. Clearly, *Xbp1* and *Atg16l1* expression in intestinal epithelial cells, and specifically Paneth cells, plays an important role in mediating intestinal homeostasis. However, given the variable effects of the microbiome on inflammation in the gastrointestinal (GI) tract [120,124], it is important to consider the interplay of host susceptibility genes and environmental factors.

## 7. Models for Studying Paneth Cells

Mice, rats, chickens, equines, nonhuman primates, and humans have Paneth cells in their small intestines, although they are not found in all animals, such as sheep, cows, and seals [3,4,127,128,129]. The prevalence of Paneth cells in animals is not fully known due to both a lack of thorough investigation and the absence of uniform criteria for identifying Paneth cells in GI tracts. Moreover, there appears to be no clear evolutionary relationship that explains the presence or absence of Paneth cells among species. Most of what we know about Paneth cell development and function is derived from mouse studies. Until recently, with the exception of limited studies of short-lived intestinal explants or crypt preparations, Paneth cells could only be studied in vivo due to a lack of a culture system. In 2009, pioneering work by the Clevers group established a new model for culturing primary intestinal epithelial cells in vitro, termed enteroids [130]. Enteroids are three-dimensional tissue culture structures that contain the diversity of intestinal epithelial cell types found in the small intestine or colon [52]. They are untransformed and can be derived from adult intestinal epithelial, embryonic, or induced pluripotent stem cells, and their method of derivation determines the nomenclature used to describe them [131]. They can also be genetically manipulated, cryopreserved, and cultured continuously for extended periods of time. The robust formation of Paneth cells in mouse enteroids allows the manipulation and investigation of these cells in vitro for the first time, and enteroids are being used extensively in recent studies of intestinal development. Paneth cells in enteroids secrete mature α-defensins that maintain antimicrobial activity, and they can be used to model oral infection by microinjecting bacteria and viruses into the lumen of enteroids, which is topologically equivalent to the small intestinal lumen [89,132,133,134]. Enteroids provide a unique opportunity to study the interaction of enteric viruses with primary, intestinal epithelial cells. They have been shown to support the replication of rotaviruses, noroviruses, enteroviruses, and adenoviruses [132,135,136,137,138]. Additionally, the utility of enteroids in investigating host-pathogen interactions has become well established.

## 8. Paneth Cell Functions In Vivo

***Mucus barrier augmentation and stem cell protection***. Unlike the colon, the small intestinal epithelium is coated by a relatively porous mucus layer that is attached loosely to epithelial cells [139]. α-defensins and other antimicrobial proteins are concentrated in the mucus to enhance the mucosal barrier [140]. The combination of antimicrobial factors and mucus makes it difficult for luminal bacteria to interact directly with intestinal epithelial cells. Additionally, because they are secreted in close proximity to the crypt base stem cells, α-defensins (estimated at 4–24 mM) and other antimicrobial factors can reach very high concentrations within the crypt lumen [79]. Therefore, they function to protect the stem cells from the microbiome or invading pathogens. 

α-defensins are potently antiviral against both enveloped and non-enveloped viruses [25,26], and the concentrations of α-defensins that are antiviral in cell culture are within the physiologic range estimated in the gut. A major mechanism whereby α-defensins inhibit enveloped viruses is by preventing viral glycoprotein interactions with their cellular receptors leading to inhibition of fusion [25,26]. In contrast, α-defensins inhibit non-enveloped viruses by binding to and stabilizing the capsid, thereby perturbing uncoating [25,26]. Direct neutralization of viral infection by α-defensins has not yet been demonstrated in vivo; however, recent studies have shown that for at least one enteric virus, mouse adenovirus 2 (MAdV-2), infection is not only resistant to neutralization by α-defensins but is actually increased or enhanced [132]. MAdV-2 is a natural pathogen of mice that infects that GI tract without causing overt disease [141]. MAdV-2 infection of traditional cell culture was increased ≈2-fold by mouse enteric α-defensins [132]. In an enteroid model, naturally secreted α-defensins increased MAdV-2 infection ≈2- to 4-fold by increasing the initial interaction of the virus with the host cell, allowing both receptor-dependent and -independent entry. Furthermore, wild-type mice infected orally with MAdV-2 shed more virus in feces than mice lacking functional enteric α-defensins. Thus, α-defensin-mediated enhancement of MAdV-2 infection occurs in two-dimensional cell culture, three-dimensional enteroid culture, and in vivo. Consistent with this finding, MAdV-2 infects Paneth cells in vivo [127]. Infection by the closely related MAdV-1 is inhibited by the same enteric α-defensins that enhance MAdV-2 infection [142], suggesting that resistance to α-defensin neutralization and the ability to utilize these host defense peptides to increase infection may be a consequence of the evolution of this fecal/orally transmitted virus under selective pressure from abundant α-defensin secretion in the mouse intestine.

Although there are only a few human adenovirus (HAdV) serotypes that are known to primarily cause gastroenteritis in humans (HAdV-12, HAdV-40, and HAdV-41), many respiratory serotypes also infect the GI tract [143,144,145]. HD5 is a potent inhibitor of only a subset of HAdVs, which cause disease outside of the GI tract [146]. The remaining serotypes of HAdV are either resistant or enhanced by HD5. These experiments were performed in a transformed lung epithelial cell line that is commonly used for studying HAdV. This effect was recently recapitulated in a more physiologically relevant system, human enteroids, which supports HAdV replication [135]. Thus, like MAdV-2 resistance to mouse enteric α-defensins, HD5 resistance of fecal/orally transmitted HAdVs may also reflect viral evolution.

Another case in which barrier integrity mediated by Paneth cells is compromised by viral infection occurs in intestinal dysbiosis following human immunodeficiency virus (HIV) infection. HIV exerts significant and detrimental effects on the GI immune system. Early studies of HIV-infected patients revealed severe enteropathy throughout the GI tract [147]. Moreover, HIV rapidly depletes CCR5^+^CD4^+^ T cells in the lamina propria following initial infection, and T cell numbers in the GI associated lymphoid tissue (GALT) do not fully recover even under effective anti-retroviral therapy [147]. This disruption may also impact epithelial integrity and function, which has been examined in a simian immunodeficiency virus (SIV) intestinal loop model. As early as 2.5 d post-infection, SIV disrupts intestinal epithelial barrier integrity [148]. Loss of epithelial integrity was accompanied by increased IL-1β expression in Paneth cells, suggesting a possible mechanism whereby Paneth cells respond to viral infection by secreting IL-1β, although the Paneth cells themselves were not infected. IL-1β in turn decreases expression of tight junction proteins, leading to epithelial permeability. In a previous study by the same group, rhesus macaques with simian AIDS (SAIDS) due to chronic SIV infection had increased numbers of Paneth cells per crypt, although the Paneth cells had reduced numbers of cytoplasmic granules [149]. Interestingly, this correlated with an increase in enteric α-defensin RNA levels but a decrease in α-defensin protein levels in Paneth cells, which was not observed in the ileal loop model [148]. The authors proposed that Paneth cells were undergoing frequent secretion in macaques with SAIDS, accounting for the absence of detectable α-defensins in Paneth cells [149]. The loss of α-defensin protein in Paneth cells correlated with an increase in bacterial and eukaryotic infections of the GI tract, suggesting that either the defensins are ineffective against pathogens in the context of SAIDS or that α-defensin protein synthesis is lost in SAIDS, resulting in reduced antimicrobial activity. Therefore, although SIV and HIV are not tropic for Paneth cells, infection by these viruses significantly impacts Paneth cell function.

***Microbiome composition***. Paneth cell antimicrobial products play a direct role in shaping the intestinal microbiome. Two mouse models have been critical in understanding the impact of α-defensins in particular on the composition of the host microbiome: *Mmp7^−^*^/*−*^ mice and *DEFA5* transgenic mice [150]. MMP7 is produced by mouse Paneth cells and converts pro-defensins into mature enteric α-defensins [49]. Thus, the *Mmp7^−^*^/*−*^ mouse is a functional α-defensin knockout in the ileum, although this is an imperfect model, because mature α-defensins that result from processing by other luminal proteases can be recovered in the caecum and colon [151]. *DEFA5* transgenic mice express HD5 at levels comparable to native mouse enteric α-defensins under the control of the *DEFA5* promoter, which restricts expression to Paneth cells [31]. *Mmp7^−^*^/*−*^ mice have an altered microbiome relative to wild-type littermate control mice with an increase in Firmicutes species and a decrease in Bacteroidetes species in the ileum [150,152]. In contrast, *DEFA5* transgenic mice had a reciprocal change with a decrease in Firmicutes and an increase in Bacteroidetes. Interestingly, while *Mmp7^−^*^/*−*^ mice were colonized by segmented filamentous bacteria (SFB), *DEFA5* transgenic mice lacked a detectable SFB population. Moreover, *DEFA5* transgenic mice and wild-type mice with low levels of SFB have fewer CD4^+^ T cells expressing IL-17A than *Mmp7^−^*^/*−*^ or wild-type mice with high levels of SFB.

The mechanism of SFB modulating T cell development in the gut has been partially elucidated. Upon SFB-intestinal epithelial cell contact, which only occurs in the ileum, type 3 innate lymphoid cells secrete IL-22, which stimulates production of epithelial serum amyloid A proteins 1 and 2 (SAA1/2) from intestinal epithelial cells [153]. It is important to note that SAA1/2 production could be due to SFB contact with intestinal epithelial cells or a combination of SFB contact and IL-22 signaling. SAA1/2 could then act directly upon Th17 cells. Thus, SFB colonization impacts Th17 effector functions in the GI tract, shaping not only the composition of the microbiome but also potentially the functionality of the GALT [150,153,154].

MNV has two strain-dependent disease profiles, causing either a persistent intestinal infection or an acute multi-organ infection [155]. Antibiotic treatment of wild-type mice infected with the persistent MNV CR6 strain completely abolished MNV replication through an interferon-λ signaling-dependent mechanism [156]. The authors hypothesized that the bacterial components of the microbiome dampened the interferon-λ signaling pathway, and antibiotic treatment relieved this dampening allowing for clearing of MNV. Although no specific component of the microbiome (i.e., LPS) was identified as important for this phenotype, the dependence of MNV infection on the presence of the enteric microbiome underscores the potential importance of Paneth cells during MNV infection in vivo. Since antimicrobial peptides secreted by Paneth cells modulate the composition of the microbiome, Paneth cells may indirectly impact MNV infection by adjusting the population of bacteria in the gut.

Paneth cells may have a similar indirect effect on poliovirus. Oral infection of mice with poliovirus requires transgenic expression of the poliovirus receptor (PVRtg) and the absence of functional interferon signaling (*Ifnar^−^*^/*−*^) [157]. Upon antibiotic treatment, fewer PVRtg/*Ifnar^−^*^/*−*^ mice succumb to poliovirus infection compared to untreated mice [157], implicating the enteric microbiome in poliovirus infection and pathogenesis. Further investigations revealed that poliovirus was thermo-stabilized by feces from conventional but not germ-free or antibiotic-treated mice and by LPS [157,158]. Moreover, poliovirus infection in vitro was enhanced by purified LPS from several different enteric bacteria, but not by other components of the small intestinal milieu (i.e., peptidoglycan, mucin). Given that Paneth cells are a critical element in shaping the microbiome, it is possible that Paneth cells indirectly impact poliovirus infection by modulating the bacterial phyla present in the small intestine.

***Innate immune sensing***. Paneth cells also play a key role in host defense by sensing microorganisms. There are numerous innate sensing pathways including inflammasomes, RIG-I-like receptors, and toll-like receptors (TLRs). The importance of TLR-mediated sensing of bacteria by Paneth cells has been specifically addressed. MyD88 is a signaling adaptor protein involved in transducing signals from TLRs, IL-1 receptor, and IL-18 receptor. Deletion of *MyD88* or expression of a dominant negative allele of *MyD88* results in decreased production of RegIIIγ, RELMβ, and RegIIIβ by the intestinal epithelium and increased susceptibility to STm [43,159]. *MyD88* expression was selectively reconstituted in Paneth cells of *MyD88^−^*^/*−*^ mice through use of the Paneth cell-specific cryptdin-2 (CR2) promoter [43]. CR2-MyD88 transgenic mice infected with STm had fewer bacteria in their mesenteric lymph node compared to infected *MyD88^−^*^/*−*^ mice, suggesting that Paneth cell intrinsic sensing and function is sufficient to restore the mucosal barrier. Interestingly, STm infection did not increase expression of the *MyD88*-dependent gene program in conventional mice, indicating that the microbiome stimulates Paneth cells to express antimicrobial genes.

Beyond a direct role in microbial sensing, Paneth cells also alter the immune response through the activities of their secreted products. α-defensins in particular function as chemoattractants [25]. HD5 induces migration of macrophages and mast cells but not immature DCs and recruits both naïve and memory T cells. Although HD5 functions as a chemokine for a variety of immune cell types, the receptor for α-defensins is unidentified. Thus, HD5 is a potent chemotactic signal for immune cells, which may also be true for enteric α-defensins from other species.

α-defensins also function to modulate the adaptive immune response. Mouse adenovirus-1 (MAdV-1) causes disease in the central nervous system, manifesting as encephalitis [141]. Additionally, MAdV-1 infection is sensitive to neutralization by mouse and human enteric α-defensins [142]. *Mmp7^−^*^/*−*^ mice are more susceptible to oral MAdV-1 challenge than WT mice; however, there was no evidence of direct enteric α-defensin antiviral activity. Rather, *Mmp7^−^*^/*−*^ mice did not develop germinal centers and had delayed neutralizing antibody responses to MAdV-1 [142]. However, *Mmp7^−^*^/*−*^ mice did not generate a sufficient antibody response to MAdV-1 infection; they were not universally impaired for humoral immunity. Intranasal challenge of wild-type and *Mmp7^−^*^/*−*^ mice with ovalbumin (OVA) resulted in development of OVA specific antibodies with similar kinetics and similar titers for both genotypes. Moreover, parenteral challenge of both wild-type and *Mmp7^−^*^/*−*^ mice with MAdV-1 abrogated any difference between the two genotypes, strongly implicating a role for α-defensins in the GI tract in generating an adaptive immune response to infection. It is possible that enteric α-defensins function as an adjuvant or in a paracrine signaling fashion to stimulate an adaptive immune response to other enteric pathogens.

## 9. Conclusions

Paneth cells play an integral part in stem cell maintenance, microbiome shaping, and host defense. Although Paneth cells were identified over a century ago, there still remains a lot to be learned about their basic biology and role in microbial infections, particularly viral infections. Although likely, it remains to be demonstrated that naturally produced enteric α-defensins either directly or indirectly impact the pathogenesis of viruses besides human and mouse adenoviruses. Other Paneth cell-derived antimicrobial peptides and proteins that shape the host intestinal microbiome also likely influence viral infection, an area that warrants further research. Enteroids have revolutionized the study of Paneth cell development and biology, allowing direct comparisons between species. Additionally, they are a reductionist system amenable to host-pathogen studies that bridge traditional cell culture and in vivo models. Studies that take advantage of the multiple platforms now available to study Paneth cells are likely to provide exciting new insights into the biology of these fascinating cells.
